# The developing wing crossvein of Drosophila melanogaster: a fascinating model for signaling and morphogenesis

**DOI:** 10.1080/19336934.2022.2040316

**Published:** 2022-03-18

**Authors:** Hanna Antson, Tambet Tõnissoo, Osamu Shimmi

**Affiliations:** a Institute of Molecular and Cell Biology, University of Tartu, Tartu, Estonia; b Institute of Biotechnology, University of Helsinki, Helsinki, Finland

**Keywords:** BMP, pattern formation, BMP binding protein, short gastrulation, crossveinless, epithelial cells, posterior crossvein

## Abstract

The *Drosophila* wing has been used as a model for studying tissue growth, morphogenesis and pattern formation. The wing veins of *Drosophila* are composed of two distinct structures, longitudinal veins and crossveins. Although positional information of longitudinal veins is largely defined in the wing imaginal disc during the larval stage, crossvein primordial cells appear to be naive until the early pupal stage. Here, we first review how wing crossveins have been investigated in the past. Then, the developmental mechanisms underlying crossvein formation are summarized. This review focuses on how a conserved trafficking mechanism of BMP ligands is utilized for crossvein formation, and how various co-factors play roles in sustaining BMP signalling. Recent findings further reveal that crossvein development serves as an excellent model to address how BMP signal and dynamic cellular processes are coupled. This comprehensive review illustrates the uniqueness, scientific value and future perspectives of wing crossvein development as a model.

## Introduction

Wing vein patterning and morphogenesis of *Drosophila* serve as an excellent model to investigate the interactions between signalling pathways during development [1,2]. In addition, due to its visibility and non-requirement for viability, the *Drosophila* wing has served as an exceptional model for phenotypic analysis [3]. In the context of wing crossvein development, Calvin Bridges reported that the *crossveinless* (*cv*) mutant is an X-linked (sex-linked) recessive allele [4]. Since then, the *cv* mutant has been used as a convenient recessive marker.

The process of crossvein development was elucidated by Conrad Waddington in 1940 [5]. Detailed observations of wing vein development indicate that the position of the posterior crossvein (PCV) is determined during the pre-pupal stage and affected by the formation of longitudinal veins (LVs). Furthermore, Waddington utilized crossveinless phenotypes to elucidate the effects of environmental perturbations [6]. Due to its convenient phenotypic analysis, crossveinless phenotypes were actively used for natural variation and the effects of environmental perturbations during the 1960s [7–14]. However, the gene responsible for crossveinless alleles remained to be elucidated until 2000.

According to ‘*The Genome of Drosophila melanogaster*’, eight crossveinless alleles have been described, including *crossveinless (cv), crossveinless-2 (cv-2), crossveinless-b (cv-b), crossveinless-c (cv-c), crossveinless-d (cv-d), crossveinless effect (cve), crossveinless like 5 (cvl-5)*, and *crossveinless like 6 (cvl-6)* [15]. Among these, the molecular mechanisms of *cv, cv-2, cv-c*, and *cv-d* have been characterized to date, and have contributed to enhance our knowledge of how crossvein development is regulated. In the following, we review molecular mechanisms behind crossvein development.

## 1. The development of wing longitudinal veins

**Figure 1.**
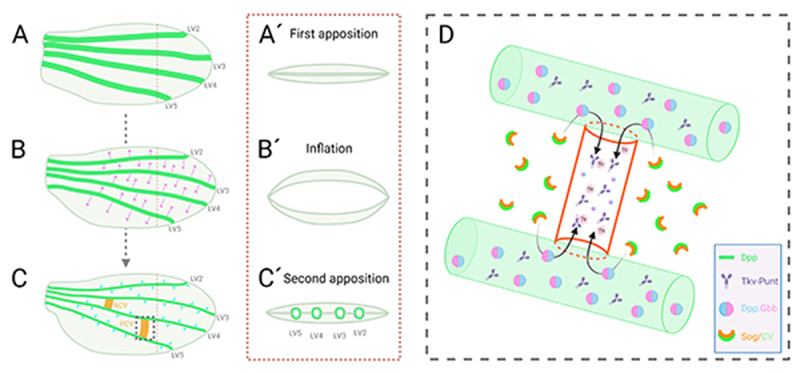
**Posterior crossvein development of the *Drosophila* pupal wing**. (A, A´) During the first apposition of the wing epithelia, around 8 h after pupariation (AP), *dpp* (green) is expressed in the longitudinal veins 2–5 (LV2-5). (B, B´) In the course of the inflation stage (10 h – 20 h AP), Dpp diffuses laterally to maintain a long-range BMP signal (magenta arrows). (C, C´) During the second apposition stage (20 h AP or later), posterior crossvein (PCV) progenitor cells are first detectable. At this stage, Dpp maintains a short-range BMP signal in the LVs (blue arrows). A long-range BMP signal is needed for PCV formation. (D) A schematic of long-range BMP signal into PCV. Sog/Cv complex facilitates Dpp:Gbb heterodimer trafficking from LVs into the PCV region. Sog is then cleaved by protease Tlr enabling the release of Dpp:Gbb heterodimers, which subsequently activates the Tkv-Punt receptor in the PCV region. Dorsal view of the pupal wing (A, B, C). Cross-section of the area shown in A, B, C with red dashed line (dorsal at the top and ventral at the bottom) (A´, B´, C´). Zoomed view of the PCV region shown in C with black dashed box (D). Created with BioRender.com.

The *Drosophila* wing is composed of two different types of veins, the LVs (LV1-5) and the crossveins (anterior crossvein (ACV) and PCV) [1,5]. During the early stage of wing development, the pattern of LVs emerges during the mid- to late third instar larval stage [1,5]. At the beginning of metamorphosis, the wing imaginal disc, a single-layered epithelial sheet, undergoes disc eversion to become a pupal wing composed of two epithelial cell layers [16,17]. During the first day of pupal development, wing formation can be divided into three distinct stages. They include the first apposition, the inflation, and the second apposition stages [4]. During the first apposition, from the start of pupariation until 10 h after pupariation (AP), proveins of LVs are formed as broad gaps between dorsal and ventral epithelia [1,5]. The provein gaps are lost during the following inflation stage between 10 h and 20 h AP, during which separation of the two wing epithelia occurs. In the course of the second apposition, starting around 20 h AP, dorsal and ventral epithelia re-appose and definitive veins become evident (Figure 1) [1,5].

In the course of wing vein formation, LVs are dependent on various highly conserved signalling pathways. They include the Notch-Delta, the Epidermal growth factor receptor (Egfr), the Bone Morphogenetic Protein (BMP), and the Rho GTPase signalling pathways [1,2,18]. They interact with and feed back into each other to initially define vein and intervein regions in the larval wing imaginal disc and refine vein patterning during pupal stages [1,19,20].

Egfr signalling is crucial for the initial specification of the LV proveins [21,22]. The pathway is vital for stimulating vein formation and impacts Notch signalling by activating the expression of *Delta* and by repressing the expression of *Notch* [23], which specifies the vein and intervein regions. The latter in turn represses the activity of Egfr signalling. Continued Egfr signalling within LVs is required to maintain vein cell fate, and later on to activate BMP signalling within the LVs by inducing the expression of the BMP-type ligand *decapentaplegic (dpp)* [21,24,25]. Dpp is secreted either as a homodimer (Dpp:Dpp) or as a heterodimer with another BMP-type ligand, Glass bottom boat (Gbb) (Dpp:Gbb) [26–28]. The ligands bind to BMP type I receptor Thickveins (Tkv) and type II receptor Punt, leading to phosphorylation of the transcription factor Mothers against dpp (Mad) [17,27,29]. Phosphorylated Mad (pMad) is then able to bind to Medea, a co-Smad, and translocates into the nucleus to regulate the transcription of various targetgenes (Table 1) [30]. BMP signalling pathway is required to maintain Egfr signalling for the continuous development of the LVs, and plays a critical role in vein specification during wing development [19,20].

**Table 1.** Molecular factors involved in the PCV development of the *Drosophila* pupal wing and their human orthologs

	*Drosophila*	Human	Similarity
BMP-type ligand	*decapentaplegic (dpp)*	*BMP2/4*	54% (*BMP2*) 57% (BMP4)
BMP-type ligand	*glass bottom boast (gbb)*	*BMP5/6/7/8a/8b*	52% (BMP7)
BMP-type I receptor	*thick veins (tkv)*	*BMPR1*	63% (BMPR1)
BMP-type II receptor	*punt (punt)*	*BMPR2*	54% (BMPR2)
BMP receptor regulated protein (R-Smad)	*Mothers against dpp (Mad)*	*SMAD1, 5, 8*	82% (SMAD1)
Common mediator Smad (Co-Smad)	*Medea (Med)*	*SMAD4*	55% (SMAD4)
BMP binding protein	*short gastrulation (sog)*	*CHRD*	40% (CHRD)
BMP binding protein	*crossveinless (cv)*	*TWSG1*	54% (TWSG1)
Protease	*tolloid-related (tlr)*	*BMP1*	66% (BMP1)
BMP binding protein	*crossveinless-2 (cv-2)*	*BMPER*	47% (BMPER)
BMP binding protein	*crossveinless (cv-d)*	ND	–
Rho GTPase-activating protein (RhoGAP)	*crossveinless-c (cv-c)*	*DLC1*	46% (DLC1)
Component of DAPC	*Dystrophin (Dys)*	*DMD*	47% (DMD)
Apicobasal polarity determinant	*scribbled (scrib)*	*SCRIB*	45% (SCRIB)
Rho family GTPase	*Cdc42*	*CDC42*	95% (CDC42)

The degree of similarity is based on protein alignment between two genes (DRSC integrative ortholog prediction tool (DIOPT) by Harvard Medical School). ND: not determined.

## 2. The development of wing crossveins

Definitive crossveins are formed after the initial specification of LVs, during the second apposition [5]. Here, we focus on mechanisms underlying PCV development, as previous studies mainly used PCV as a model. Immediately prior to the second apposition stage, PCV between L4 and L5 first becomes detectable around 18 h AP as a broad zone of crossvein progenitor cells [5,21,25]. As PCV development progresses between 20 h and 28 h AP, the broad zone of the PCV field starts to constrict, leading to the formation of a narrow, refined and well-defined PCV [21,31,32].

The specification of the PCV has been shown to be regulated by induction of BMP signalling [21,25,33]. During the pupal stage, *dpp* is expressed in the LVs but not PCV prior to 28 h AP. However, BMP signal is detectable in all wing progenitor cells, including crossveins, at 18 h AP or later, indicating that long-range BMP signalling is required for PCV formation until *dpp* itself is expressed in the PCV (Figure 1C) [21,26]. Visualizing functional GFP-tagged Dpp in the pupal wing reveals that Dpp-GFP moves from the LVs into the PCV field [26]. In contrast, most Dpp-GFP is immobilized near the LVs to maintain a short-range signalling (Figure 1C, D) [26]. These data indicate that both short-range BMP signalling in LVs and long-range BMP signalling towards the PCV are required for wing vein patterning.

One group of *dpp* mutant alleles called *shortvein (shv)* contains mutations in the enhancer region responsible for *dpp* transcription during the pupal stage [34,35]. Since *dpp^shv^* mutant flies display a loss of wing veins distally, Dpp has been considered to serve as a wing vein determinant during the pupal stage. Recent studies, however, reveal that BMP signalling coordinates both tissue growth and vein patterning during pupal wing development [16]. This is evidenced by the fact that a conditional knockout of *dpp* in the pupal wing leads to smaller wing size and loss of vein formation. Dpp forms a long-range BMP signal by maintaining lateral trafficking of ligands during the inflation stage at the time when active proliferation occurs (Figure 1B, B´) [16]. Then BMP signal switches over to short-range signalling around the period of the second apposition (~18 h AP) to function as a wing vein determinant (Figure 1C, C´). The change in BMP signalling range coincides with changes in the three-dimensional wing structure [16]. Interestingly, when the dorsal and ventral wing epithelia re-appose, BMP laterally signals over a short range, but also maintains active vertical communication between the two epithelial layers. This may help the final refinement of wing vein patterning. Thus, the long-range BMP signal in the PCV region starts taking place when the wing structure changes from inflation to the second apposition stages.

## 3. Long-range BMP signalling during PCV development

How is a long-range BMP signal generated in the PCV field, even though the BMP signal changes to short-range in the LVs? The existence of a class of genes needed for crossvein formation in *the Drosophila* wing provides clues to understand the mechanisms. Genetic analysis has revealed that several components are required for BMP signalling in the PCV region. These include two BMP-type ligands, Dpp and Gbb, and two BMP binding proteins, short gastrulation (Sog) and crossveinless (Cv) (Table 1).

In addition to Dpp, Gbb is required for PCV formation [25,27,28,36]. Both Dpp and Gbb ligands move from LVs into the PCV region from 18 h to 28 h AP [21,26,27]. The spatial distribution of BMP ligands is tightly regulated at the extracellular level. Since a short-range BMP signal is observed in LVs during this period, mechanisms that move ligands into PCV are needed.

**Figure 2.**
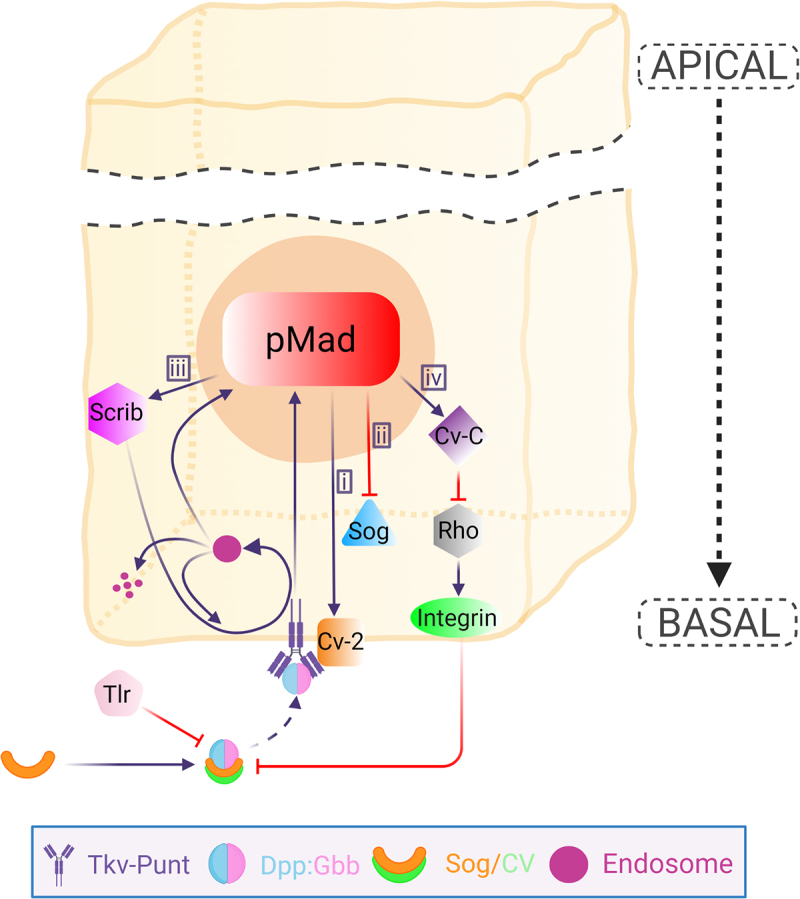
**Schematic overview of BMP signalling regulatory system in the PCV cell**. The Dpp:Gbb heterodimer is trafficked basally with BMP binding proteins Sog/Cv to bind Tkv-Punt receptor. The release of the ligands requires protease Tlr that is responsible for the cleavage of Sog. The release of the heterodimers leads to the activation of the receptor prompting BMP signalling highlighted by pMad expression. BMP signal regulates various co-factors that form a feedback or feedforward mechanism to further sustain BMP signal in the PCV field. i) Cv-2 is upregulated by BMP signal. Cv-2 is a secreted BMP-binding protein that promotes BMP signalling by facilitating receptor-ligand binding. ii) BMP signal represses *sog* expression which is needed for continuous PCV formation. iii) BMP signal is needed for up-regulating Scrib expression, which in turn optimizes the BMP signalling by regulating the localization of Tkv and by facilitating Tkv internalization to Rab5 endosomes enabling the receptor signalling. The endosomes can either enter into the receptor recycling or go to degradation. iv) BMP signal induces *cv-c* expression, which is responsible for inactivating Rho-type small GTPases, leading to the downregulation of β-integrins. Low levels of β-integrins provide optimal extracellular environment to maintain ligand trafficking into the PCV region. Apical portion of the epithelial cell is abbreviated. Created with BioRender.com.

Two BMP binding proteins, Sog and Cv, play such a role by forming a complex with and facilitating the long-range directional transport of Dpp and Gbb from the LVs towards the PCV field (Figure 1D) [26,27]. Sog, an ortholog of Chordin in vertebrates, was originally identified as a secreted BMP binding protein needed for dorsoventral axis formation in the early embryo [37,38]. Later, Sog was also found to be needed for BMP signalling in the PCV field of the pupal wing [21,39]. Sog inhibits BMP signalling by binding to and sequestering BMP ligands, preventing the activation of receptors (Figure 2) [39,40]. Interestingly, the absence of *sog* transcription appears to prepattern the PCV position [21,26]. During 18 h to 20 h AP, *sog* expression is repressed in the PCV field in a BMP signal-independent manner to provide the positional information of the BMP signal. At later stages of PCV development, continuous BMP signal is needed for maintaining the repression of *sog* transcription by forming a feedback loop (Figure 2) [26]. It remains to be addressed how BMP signal-independent *sog* expression is regulated during PCV development.

Secreted protein Crossveinless (Cv) is functionally equivalent to Twisted gastrulation (Tsg), another dorsoventral determinant in the embryo [27,41,42]. Cv was originally named Tsg2 but later identified to encode the gene product of the *cv* allele. A Sog-Tsg complex leads to facilitated transport of a BMP heterodimer comprising Dpp and Screw, another BMP type ligand, for BMP morphogen gradient formation in the embryo [43,44]. Tolloid (Tld) protease is then needed for BMP ligand binding to the receptors through releasing ligands from the Sog-Tsg complex after cleavage of Sog [45]. During pupal wing development, Tolloid related (Tlr), a paralog to Tld, plays such a role [46]. Similar to Sog, Tlr is expressed in the intervein regions of developing wings, indicating its role in the arrangement and refinement of vein formation. As *tlr* mutants exhibit a PCV-less adult wing phenotype, the protease is shown to play a role in the specification of the PCV. Moreover, Tlr is needed to ensure localized and high levels of BMP signalling during PCV specification by cleaving and inactivating the BMP binding protein Sog [46]. This leads to the release of ligands to activate the receptors in the PCV to accomplish restricted patterns of signalling in the pupal wing [33,46]. Thus, PCV formation needs a facilitated transport mechanism of BMP ligands, in which Dpp:Gbb heterodimer produced in the LVs is moved into the PCV field by a Sog-Cv complex, and then Dpp:Gbb ligands are released from the Sog-Cv complex via the activity of Tlr to bind to the BMP-type receptors Tkv and Punt for signalling Figure 1D).

The expression of BMP type I receptor Tkv in the LVs is crucial for maintaining vein width by regulating the BMP signalling range in the LVs [21,24]. Dpp ligands are actively retained in the LVs by Tkv-ligand interactions and a feedback mechanism [26,47]. Since ectopic expression of a constitutive active form of Tkv (Tkv^QD^) in the LVs leads to loss of BMP signal in the PCV field, a feedback mechanism appears to be crucial for PCV development as well [26]. As Dpp:Gbb heterodimers are intrinsically retained in the LVs by Tkv, the Sog-Cv-mediated transport is thought to liberate Dpp:Gbb heterodimers from the positive feedback mediated retention in the LVs, thus enabling PCV formation (Figure 1D) [26,27,33]. Precise molecular mechanisms of the feedback loop remain to be identified.

## 4. Co-factors involved in PCV development

During early PCV development, Dpp ligand observed in the PCV field is limited, even though pMad signal, a readout of the BMP signal, is robust [16,26] (Vi Tran et al., unpublished data). Therefore, co-factors may play a crucial role in sustaining BMP signalling in the PCV field. Next, we review co-factors responsible for regulating BMP signalling in the PCV field (Table 1).

### Crossveinless-2

Crossveinless-2 (Cv-2) is required for boosting BMP signal in the PCV region [48]. Cv-2 is composed of two domains: an N-terminal cysteine-rich domain and a C-terminal von Willebrand Factor domain [25]. Cv-2 is a secreted BMP-binding protein that enhances or inhibits BMP signalling in a context-dependent manner and loss of Cv-2 function leads to a PCV-less phenotype [25,48]. *cv-2* expression in the PCV region is regulated by BMP signalling, and thus Cv-2 is a part of a positive feedback loop (Figure 2). It is highly expressed in the PCV and serves to promote high levels of BMP signalling in the area through facilitating receptor-ligand binding [25,48].

### Crossveinless-d

One of the co-factors needed for proper BMP signalling during PCV morphogenesis is Crossveinless-d (Cv-d) [49]. It is a vitellogenin-like lipoprotein produced in fat bodies and other tissues and is able to diffuse into the pupal wing via the hemolymph [47]. Cv-d binds BMP ligands Dpp and Gbb through its Vg domain. As BMP binding to Vg domain is necessary but not sufficient for Cv-d activity, lipid binding is also proposed to be necessary for Cv-d function. Cv-d accumulation and PCV development require Heparan Sulphate Proteoglycans (HSPGs) [49]. As Cv-d acts over a long range, it has been suggested that Cv-d affects the range of BMP movement in the pupal wing, probably as part of a lipid-BMP-lipoprotein complex. As more severe PCV defects can be observed at later pupal stages, Cv-d is also important for maintaining BMP signalling in addition to its extracellular role in regulating BMP signalling in the PCV during initial stages of PCV development [49].

### Dystrophin

Dystrophin and Dystroglycan are two central components of the Dystrophin Associated Protein Complex (DAPC) [50]. In *Drosophila, Dystrophin (Dys)* encodes the ortholog of human Dystrophin, and the mutations of *Dys^det^* lead to variable defects in the formation of PCV, ranging from mild to severe [15,31]. As null mutations in Dystroglycan (Dg) produce a phenotype similar to that of *Dys^det^*, a link between the molecular function of the DAPC and its involvement in the PCV specification has been suggested [31]. A model has been proposed by which during the early stage of PCV development, BMP signal activates the DAPC in the PCV region. DAPC then downregulates Notch signalling to antagonize BMP signalling, where the diffusion of Dpp:Gbb heterodimers is disrupted into the presumptive PCV region from LV4 and LV5 [31].

## 5. Co-factors coupling BMP signal and wing morphogenesis in the PCV region

When BMP signal instructs vein fate in the PCV field, wing vein morphogenesis represented by apical constriction and lumen formation takes place [32,33]. Due to its relatively simple structure and well-defined molecular mechanisms, PCV development serves as an excellent model to address how extrinsic cues and tissue morphogenesis are coordinated during dynamic tissue development [51]. In the following section, we review in greater detail how cellular signal is required for PCV development and how BMP signal and wing morphogenesis are coupled.

### Crossveinless-C

Crossveinless-C (Cv-C), originally found in 1934 [15], was shown to encode the Rho GTPase-activating protein RhoGAP88C [52]. Cv-C is the key molecule coordinating BMP ligand trafficking and PCV morphogenesis. Cv-C is induced at PCV primordial cells by BMP signalling, where it cell-autonomously inactivates signalling from several Rho-type small GTPases, including Cdc42, Rac1, Rac2 and Rho1. This leads to cell-autonomous downregulation of Rho GTPase targets, such as integrins [33]. Cv-C is also required for BMP ligand trafficking into the PCV region in a non-autonomous manner. The downregulation of integrins in the basal compartment of the PCV field provides an optimal extracellular environment for BMP ligand trafficking. The cellular distribution of integrins is a key regulator for extracellular BMP ligand movement [53]. BMP ligands then preferentially accumulate on the basal side of the PCV region [26]. Therefore, these cellular mechanisms mediate a feed-forward loop that coordinates BMP ligand trafficking and PCV morphogenesis (Figure 2) [33]. These mechanisms not only play a role in achieving precise tissue morphogenesis but also may be used for producing variations of crossveins. Ectopic crossvein formations are often observed in mutant alleles of *Cdc42* [54]. Loss of Cdc42 causes ectopic BMP signal in the intervein regions during the early pupal stages, resulting in ectopic crossvein formation [33]. Thus, loss of Rho-type GTPase signal has an instructive role in generating crossveins.

### Scribble

The Scribble complex is one of the key molecules responsible for establishing apicobasal polarity in epithelial cells and plays a crucial role in tissue homeostasis [55–57]. The complex is composed of Scribble (Scrib), Discs large 1 (DLG1), and Lethal giant larvae (LGL) proteins [56]. The complex has been identified to be required for PCV development as well since knockdown of *scrib, dlg1*, or *lgl* leads to defects in PCV formation [32]. Scrib is needed for optimizing BMP signal in the PCV region in a cell-autonomous manner. Scrib and BMP signal form a positive feedback loop to maintain BMP signals during the PCV morphogenesis in the pupal wing (Figure 2) [32]. BMP signal increases the Scrib levels for further polarization, which then facilitates basal accumulation of the BMP type I receptor Tkv where BMP ligand trafficking takes place (Figure 2) [33]. Scrib also facilitates Tkv internalization to the Rab5 endosomes that plays an important role in BMP signal transduction. Thereby, Scrib is needed not only for sustaining epithelial polarity but also for maintaining BMP signalling during PCV morphogenesis. These findings illustrate how BMP signal and cellular mechanisms are coordinated in PCV development.

## 6. Perspectives

Here, we summarized current knowledge about how wing crossveins have been studied in the past, and how the analysis of classical mutant alleles led to the unveiling of molecular mechanisms of PCV development. One of the classical alleles *cv* has been used as a recessive marker [4], however it remained unknown what molecular mechanisms are required for crossvein formation. Interestingly, after finding one of the dorsal patterning genes *tsg* [58], a Tsg paralog was identified and named Tsg2 [41]. Since *tsg2* proved to be allelic to the *cv* allele and Tsg2 can rescue *cv* mutants, *cv* encodes this Tsg-like protein [27,42]. Due to the similarity of components needed for dorsal patterning in the embryo and PCV formation in the pupal wing, a trafficking mechanism of BMP ligands has been proposed for PCV formation [27,42]. Since the deep homology of conserved Chordin/Sog-mediated BMP trafficking has been hypothesized [59], the system that mobilizes BMP ligands by Sog complex appears to be co-opted for PCV development. A trafficking mechanism is not only used for PCV formation of the *Drosophila* wing but also in wing vein development in other insect species as well [60–62].

Studies of PCV development highlight the roles of unique co-factors required for BMP signalling in the PCV development (Table 1). Although the molecular mechanisms behind PCV formation have been thoroughly investigated, questions remain that need to be addressed in the future.

First, understanding the pre-pattern information of crossveins is important. Previous studies revealed that loss of *sog* transcription in the PCV field instructs where BMP signal is induced [26]. However, how *sog* transcription is regulated prior to BMP signal induction is unknown. One intriguing observation relevant to this question is that weak alleles of *cdc42* often lead to ectopic crossveins by inducing Sog-mediated BMP signal [26]. Transcriptional control of RhoGTPase regulators, e.g. Rho guanine nucleotide exchange factors (RhoGEFs), may be involved in pre-pattern information. Ectopic crossvein phenotypes are also observed in other contexts, e.g. ectopic expression of transglutaminases [63,64].

Second, further elucidation of the feedback mechanism found in the LVs is crucial. The ectopic expression of constitutively active Tkv in the LVs disrupts BMP signal in the PCV field [26]. This may suggest that BMP ligands become immobilized and are unable to move into the PCV field. Contrary to this, ligands in the LVs acquire the mobility to generate a wider range of signal when the feedback mechanism is disrupted, leading to failure of proper wing vein patterning [26]. As the molecular components involved in the feedback mechanism are largely uncharacterized, future studies will be needed to understand how short- and long-range signals are regulated for wing vein patterning.

Finally, previous studies revealed that the PCV serves as an excellent model to address how BMP signal and tissue morphogenesis are coupled [32,33]. To further understand how BMP signal and dynamic cellular changes are coordinated, employing in vivo live imaging may play a key role. Recent studies reported a live imaging protocol to observe PCV morphogenesis in vivo [65]. Extension of this line of research will be important in the future.
